# Menstrual cycle characteristics of young females with occult primary ovarian insufficiency at initial diagnosis and one-year follow-up with serum amh level and antral follicle count

**DOI:** 10.1371/journal.pone.0188334

**Published:** 2017-11-27

**Authors:** Yilmaz Guzel, Yilda Arzu Aba, Kayhan Yakin, Ozgur Oktem

**Affiliations:** 1 Istanbul Aydin University, Department of Obstetrics and Gynecology, Istanbul, Turkey; 2 Istanbul Aydin University, School of Health Sciences, Istanbul, Turkey; 3 American Hospital Women’s Health Center Assisted Reproduction Unit, Istanbul, Turkey; 4 Koc University School of Medicine, Department of Obstetrics and Gynecology, the Division Reproductive Endocrinology and Infertility, Istanbul, Turkey; University of Kansas Medical Center, UNITED STATES

## Abstract

Occult primary ovarian insufficiency (also known as incipient ovarian failure or diminished ovarian reserve) is defined as serum AMH level ≤1.1ng/mL in women under age 30. Limited data is available regarding the prevalence of occult POI, the preceding menstrual characteristics and its natural course in otherwise healthy young females. We aimed in this prospective observational study to determine the prevalence of occult POI in young females (< age 30) screened with serum AMH measurement; and analyze the patterns of change in their menstruation at initial assessment and one-year follow-up in relation to the changes in ovarian reserve quantitatively assessed with AMH and AFC. 963 young female college students under age 30 voluntarily participated in this study. 43 of them (4.4%) were diagnosed with occult POI as their AMH levels were ≤ 1.1ng/mL. Thirty-eight (83.4%) of them have regular cycles and denied any menstrual irregularity in the last 12 months. This rate was not statistically different from 7.3% of those with AMH>1.1ng/mL who reported at least one abnormal menstrual cycle in the last year (p = 0.36). Cycle length was significantly shorter in females with AMH ≤ 1.1ng/mL compared to those with AMH>1.1ng/mL (25.1±3.2 vs. 31.2±2.8 respectively, p<0.001). Karyotype, FMR-1 mutation analyses and auto-antibody screening returned normal in all. At one-year follow-up AMH, AFC and mean cycle length were further reduced compared to their values at initial assessment. Now, a greater proportion of the participants with occult POI were menstruating regularly at every 21 days compared to the initial evaluation one year ago (39.5% vs. 13.9% respectively, p = 0.013). Twenty-five underwent oocyte cryopreservation. These findings underscore the importance of screening young females with AMH for possible occult POI. It also emphasizes that young females with critically diminished ovarian reserve may continue to menstruate regularly without any characteristic menstrual abnormality other than shortening of cycle length.

## Introduction

Primary ovarian insufficiency (POI) is a condition in which women under the age of 40 experience oligomenorrhea or amenorrhea for 4 months or more, in association with serum follicle stimulating hormone (FSH) levels in the menopausal range on two occasions obtained at least one month apart according to the most recent ESHRE guidelines [1, 2]. POI affects 1 in 10,000 women by age 20, 1 in 1000 by age 30, and 1 in 100 by age 40 [3]. The term primary ovarian insufficiency appears to be a more appropriate to describe the condition that was previously referred to as premature menopause or premature ovarian failure because it differs from menopause in that there is a varying degree of residual ovarian function in nearly half of the cases allowing 5–10% of women to conceive and deliver a child [2]. In fact, the term POI describes a continuum of impaired ovarian function rather than a specific end point of permanent loss of ovarian function. It is also less stigmatizing than the other terms premature ovarian failure or premature menopause [4, 5].

Occult form of POI was initially described as the triad of infertility, regular menses and elevated plasma FSH concentrations [6]. There is not an established serum AMH level to identify women with occult POI. But, AMH level (<1.1ng/mL) constitutes one of the Bologna criteria developed to define poor responding IVF patients as an hormone marker of diminished ovarian reserve (DOR) [7]. AMH level<1.1ng/ml in women ≤ age 30 was used as an indicator of DOR in infertile populations in other studies as well [8, 9]. The diagnosis of occult POI is problematic as it may develop insidiously without development of oligo/amenorrhea and there is no a proper diagnostic method in its early recognition as mentioned above. This is particularly true for young females who are otherwise healthy, not interested in childbearing and have regular menses. Thus, any subfertility problem in these women might be hidden and occult POI as an underlying cause of it may escape from professional attention and remain undiagnosed until it progresses into more advanced stages of low ovarian function and finally becomes symptomatic as menstrual abnormality. Limited data is available in the literature regarding the prevalence of occult POI, the preceding menstrual characteristics and its natural course in otherwise healthy young females. We therefore aimed in this prospective observational study to determine the prevalence of occult POI in young females (< age 30) screened with serum AMH measurement; and analyze the patterns of change in their menstruation at initial assessment and at one-year follow-up in relation to quantitative assessment of ovarian reserve with AMH and AFC.

## Patients and methods

### Patients

The study was approved by the Institutional Review Board of Istanbul Aydin University (IRB#: B.30.2.AYD.0.00.00–480.2/018). Since this study aims to identify young females with occult POI (defined as serum AMH level ≤ 1.1 ng/mL in females under age 30) all students under age 30 were invited to participate in the study using flyers in the main campus of Istanbul Aydin University. There were no pre-selection criteria for inclusion in the study. All participants who were within the required age range were evaluated regardless of their menstrual pattern. There were some exclusion criteria were as follows: the presence of metabolic-endocrine diseases, polycystic ovarian syndrome (PCOS), endometriosis and/or endometriomas, current or past use of oral contraceptive pills (OCP) or other drugs that may affect ovulatory function and hypothalamic-pituitary-ovarian axis (HPO), history of exposure to gonadotoxic chemotherapy regimens and/or radiation for malignant or non-malignant conditions, history of ovarian diseases or surgery, unilateral or partial oophorectomy. A total of 963 young female college students under age 30 voluntarily participated in this study after excluding 18 for OCP use (n = 4), PCOS (n = 12), history of surgical removal of unilateral endometrioma (n = 1), Hodgkin lymphoma survivor previously exposed to cyclophosphamide (n = 1). All participants gave informed consent. They were asked to fill a questionnaire regarding the status of their menstrual cycles, menstrual symptoms, life style, exercise, contraceptive use, personal and family history of endocrine and metabolic diseases, and premature ovarian failure (POF) (S1 and S2 Tables (English and Turkish versions, respectively). The participants were first given information about normal patterns and length of menstrual cycle and its physiological variations that can erroneously be considered as abnormal pattern by the participants. Then, type of menstrual irregularity if any, was further detailed in the questionnaire and interviewed by the authors Y.G. and/or Y.A.A at the completion of the questionnaire. These authors have experience in the definitions and diagnoses of normal and abnormal menstrual patterns. Serum samples for hormone measurements were obtained by venipuncture and stored at -80°C until assayed. Those with AMH level ≤ 1.1 ng/mL were called for further workup and scheduled for a second visit at early follicular phase of the next cycle.

In the second visit AMH test was repeated along with measurement of FSH, LH, E_2_, anti-thyroid (anti-peroxidase and anti-thyroglobulin) and anti-adrenal (21-hydroxylase, CYP-21) antibodies in the blood samples at early follicular phase. Antral follicle counts (AFC) (2-10mm) were determined, and ovarian pathology (such as endometrioma and dermoid cyst) were ruled out using ultrasound exam with Voluson E8 (General Electric Health Care, Istanbul, Turkiye) transvaginal or transabdominal probes. Transvaginal ultrasound was not performed in 4 out of 43 (9.3%) participants with occult POI due to their virginity. In these cases, transabdominal approach was performed. The ultrasonographers were blind to the AMH results. Genetic work up was also done for karyotype analysis and pre-mutation (61–199 CGG repeats) and intermediate (41–60 repeats) alleles in the FMR-1 gene. The participants were given a menstrual record chart to keep the records of menstrual cycles until the next visit one year later (no. of days from start of period to beginning of next, total duration of bleeding for each period, no. of pads used for each period, type of flow (exceptionally light, normal, exceptionally heavy, spotting), any abnormal pattern of bleeding (start day of cycle, duration, type of flow, associated symptoms, recurrence)(S3 and S4 Tables, English and Turkish versions respectively). In the third visit, one-year medical and surgical history and medications were questioned. AMH, FSH, LH, E2 and AFC were determined at early follicular phase. The cycle characteristics were obtained and mean cycle length was calculated after reviewing the records of the participant. There were no cases lost to follow-up.

### Hormone assays

Serum samples were collected via venipuncture in the antecubital veins and stored in -80°C until assayed. Electrochemiluminescence immunoassay (ECLIA, Cobas) kits specific for estradiol (Elecsys^®^ Estradiol II), FSH (Elecsys^®^ FSH) and LH (Elecsys^®^ LH) were used according to manufacturers’s instructions. The lower detection limits for E_2,_ FSH and LH were 5.0 pg/mL, 0.100 mIU/mL, and 0.100 mIU/mL, respectively. All analyses were performed on Cobas^®^ 6000 analyzer series (Roche Diagnostics). AMH levels were determined using Active Mullerian Inhibiting Substance/ Anti-Mullerian Hormone (MIS/AMH) (Diagnostic Systems Laboratories, Inc., USA) ELISA kit. The analytical sensitivity of the kit was 0.006 ng/mL. Intra-assay repeatability and the coefficient of variations were given as 4.6% (0.144 ng/mL) and 2.4% (0.843 ng/mL), respectively.

### FMR1 repeat length

DNA isolated from peripheral blood lymphocytes by conventional techniques was used to determine the FMR1 CGG repeat length size in the participants diagnosed with occult POI. The repeat region was PCR amplified with fluorescently labeled primers and sized by capillary electrophoresis with confirmation of positives with Southern blot analysis. The following criteria suggested by ACOG was used to define the alleles: %40 repeats as normal, of 41–60 repeats as intermediate, of 61–199 repeats as pre-mutation, and of >200 repeats as full mutation alleles [10].

### Statistical analysis

The data base of the all participants and those with occult POI were provided as SPSS data (S1 and S2 Informations, respectively). Continuous data were expressed as the mean±SD and compared between the participants with and without occult POI using independent student t-test or Mann-Whitney-U test according to the distribution of the data. Categorical variables were compared using Fisher’s exact test. Two-tailed Pearson correlation test and linear and non-linear regression models were applied to explain the association of AMH with age. Paired t test was utilized to compare the continuous parameters of the participants with occult POI between the initial and one-year follow-up evaluations. The significance level was set at 5% (p< 0.05). SPSS statistical programs (version 23) were used to analyse the data and create the figures.

## Results

Demographic characteristics of the 963 participants are summarized in the Table 1. In brief, the mean±SD of age and AMH of the participants were 20.1±2.4 and 3.7±1.8ng/mL respectively. 43 of them (4.5%) had serum AMH levels ≤ 1.1ng/mL. These cases therefore were diagnosed as having occult primary ovarian insufficiency (POI) according to the previous classification [8]. The remaining 920 participants had AMH levels>1.1ng/mL. The mean age, BMI, day of menstrual cycle at the time of blood sampling, and age at menarche were comparable between the participants with and without occult POI (Table 1).

**Table 1:** Baseline demographic characteristics of the participants with and without occult POI. The mean age, BMI, day of menstrual cycle at the time of blood sampling, and age at menarche were comparable between the participants with and without occult primary ovarian insufficiency (POI). The mean cycle length reported by the participants with occult POI were significantly shorter than those without it. Significantly higher number of participants with occult POI reported a positive family history of premature menopause in their mothers and/or sisters compared to those without occult POI.

	Overall	AMH≤1.1ng/mL	AMH>1.1ng/mL	P
N	963	43	920	
Age				
Mean±SD	20.1±2.4	21.1±2.8	20.1±2.3	NS
Range	17–29	17–29	17–29	
AMH				
Mean±SD	3.7±1.8	0.83±0.2	3.89±1.7	0.0001
Median	3.4	0.90	3.5	
Range	0.3–8.9	0.3–1.1	1.2–8.9	
Quartiles				
25	2.38	0.62	2.55	
50	3.44	0.90	3.55	
75	4.87	1.03	4.93	
BMI				
Mean±SD	21.6±3.8	22.5±4.4	21.2±2.8	NS
Age at menarche				
Mean±SD	13.1±1.2	13.3±1.4	13.1±1.2	NS
Cycle length				
Mean±SD	30.9±3.1	25.1±3.2	31.2±2.8	0.01
Cycle day at assessment				
Mean±SD	13.1±1.2	12.6±2.2	14.9±1.5	NS
At least one episode of irregular menses in the last 12 months	73 (7.5%)	5/43 (11.6%)	68/920 (7.3%)	NS
Family history of premature menopause before age 40				
Mother	67 (6.9%)	7 (16.3%)	60 (6.5%)	0.024
Sister	10 (10.4%)	2 (4.6%)	6 (0.7%)	0.046
Aunt	26 (2.7%)	0	26 (2.8%)	NS

AMH: Anti-mullerian hormone

BMI: Body mass index

### Quadratic regression model fits the decline in AMH with age better than linear type

**Fig 1:**
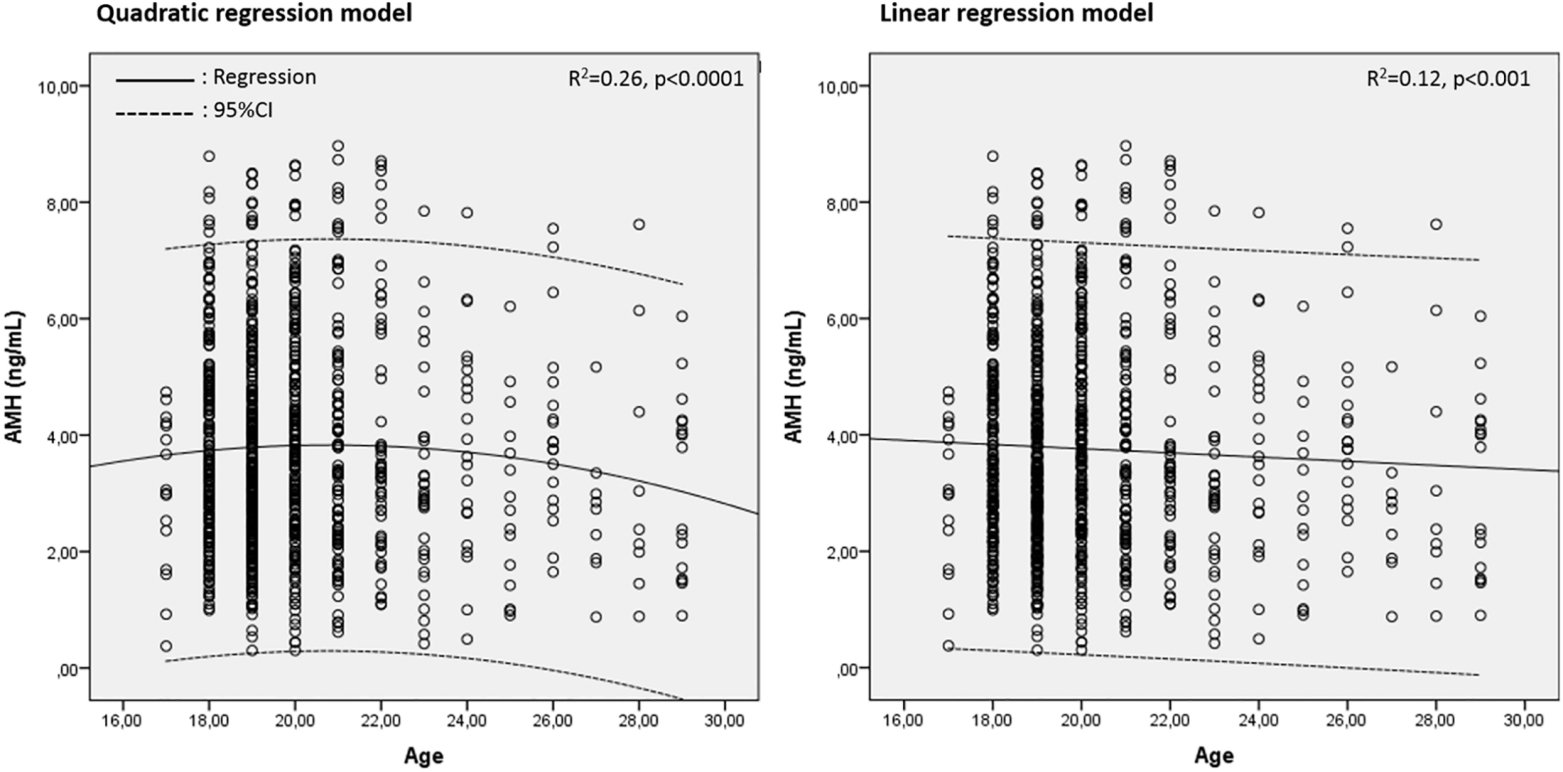
There was an inverse relation between age and AMH. Quadratic regression model explains this relation better than linear type.

There was an inverse relation between age and serum AMH level on linear and non-linear regression models when all participants were included. Based on the adjusted R^2^ statistics, non-linear quadratic regression model (R^2^ = 0.26, p<0.0001) explained this association more optimally than does a linear type regression model (R^2^ = 0.12, p<0.001) (Fig 1). This is in line with the results of the previous studies showing that quadratic regression model fits better the decline in AMH with age than linear type [8, 11, 12].

### There is no a specific pattern of menstrual abnormality reported by females with occult POI other than shortening of the cycles

**Fig 2:**
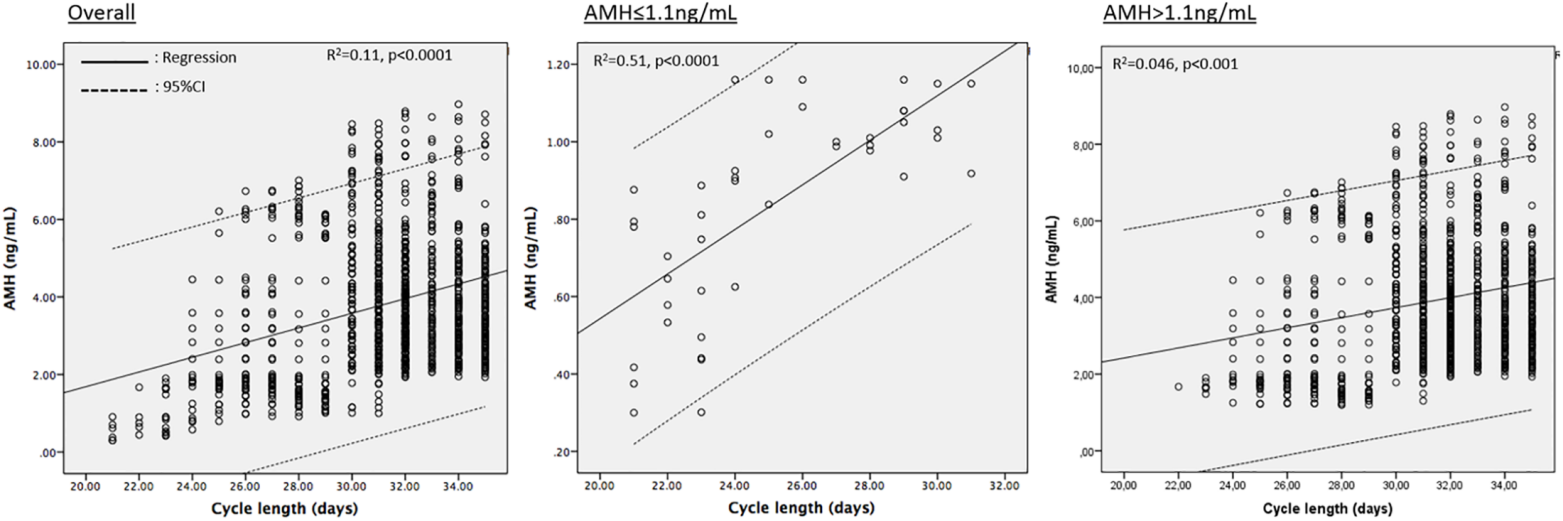
There was a positive association with AMH level and cycle length on the linear regression analyses. The association was more significant for the participants with occult POI compared to those without it.

At initial assessment, thirty-eight (88.4%) of the females diagnosed with occult POI denied any menstrual irregularity in the last 12 months including those with more drastically diminished ovarian reserve (AMH<0.5ng/mL). The remaining five (11.6%) reported mid-cycle spotting (n = 2) and spotting before menstruation (n = 3). This rate was no different from 7.3% (68/920) of those with AMH>1.1ng/mL who reported at least one abnormal non-specific menstrual cycle in the last year (p = 0.36). However, when the cycle length was questioned, it appeared that significantly higher proportion of females with occult POI were complaining of shortening of their cycles in the last twelve months compared to those without occult POI (60.4% vs. 4.4% respectively, p<0.0001). The cycle length was found to be significantly shorter in the participants with occult POI compared to those without it (25.1±3.2 vs. 31.2±2.8 days respectively, p<0.01)(Table 1). Overall, there was a positive association with AMH level and cycle length on the correlation (r = 0.32, p<0.01) and linear regression analyses (R^2^ = 0.11, p<0.001). This association was more significant for the participants with occult POI (R^2^ = 0.52, p<0.0001) compared to those without it (R^2^ = 0.046, p<0.01) (Fig 2). 26 of 43 participants (60.4%) with occult POI had a cycle length ≤25 days compared to only 41 of 920 participants without it (4.4%, p<0.0001). 6 out of 43 with occult POI (13.9%) were menstruating regularly at every 21 days and did not report any menstrual abnormality in the last year other than shorter cycles despite their critically decreased AMH level (the mean±SD: 0.59±0.2 range: 0.3–0.8 ng/mL). By contrast, none of the participants with AMH>1.1ng/mL had a cycle length of ≤ 21 days (p<0.0001). Significantly higher number of participants with AMH≤1.1ng/ml reported a positive family history of POF in their mothers and/or sisters compared to those with AMH>1.1ng/mL (20.9% vs. 7.2% respectively, p<0.01) (Table 1).

### Follow-up visits

**Fig 3:**
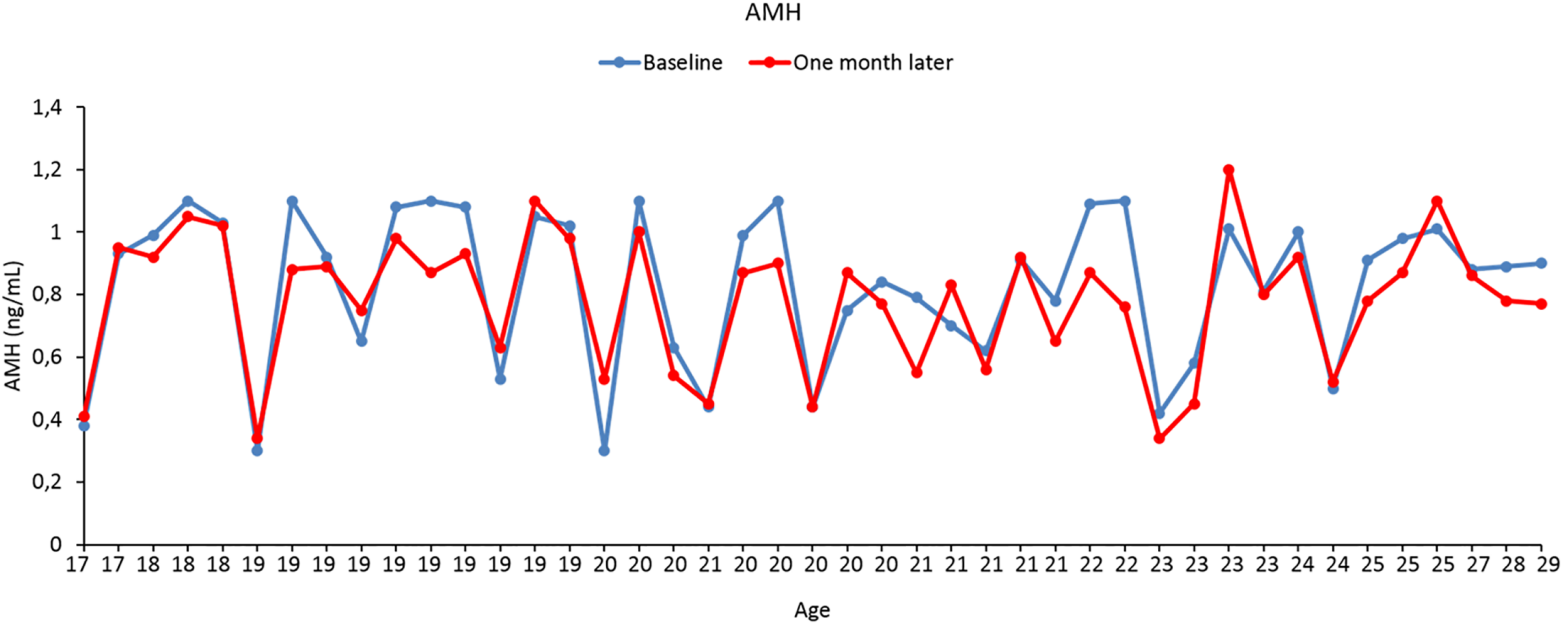
Repeat AMH measurements in the second visit one month later at early follicular phase were comparable to those at initial assessment in the participants with occult POI.

The participants who had been diagnosed with occult POI in the initial assessment were re-evaluated in a second visit 1.3±0.7 months later. Repeat AMH measurements at early follicular phase (0.78±0.2ng/mL range: 0.3–1.2) were comparable to the first readings (0.83±0.2ng/mL range: 0.33–1.1, p = 0.33) (Table 2, Fig 3). There was only one participant, whose AMH level increased from 1.01 at initial assessment to 1.2 in the second visit, exceeding the threshold level of 1.1 ng/mL. The mean antral follicle count (2.4±0.9) was significantly associated with AMH level (R^2^ = 0.19, p = 0.003). Only two participants had elevated FSH (>25 mIU/mL) at early follicular phase with the remaining having normal FSH, LH and E_2_ levels (Table 2). Anti-thyroid and anti-adrenal antibodies were within their normal ranges (data not shown). Karyotype analysis was 46, XX and FMR-1 mutation screening returned normal in all of the 43 participants diagnosed with occult POI.

**Table 2:** Menstrual cycle characteristics, and the mean levels of AMH, AFC and FSH of the participants diagnosed with occult POI at initial assessment and one-year follow up. Serum AMH level and AFC were significantly decreased and FSH level was significantly increased at one-year follow-up of the participants with occult POI compared to their corresponding values at initial assessment. The mean cycle length was significantly shortened and the number of the participants menstruating every 21 days were significantly increased compared to the initial assessment. While none of the participants diagnosed with occult POI had reported any skipped menses in the first assessment, now six of them reported one missed period at one-year follow-up.

	AMH≤1.1ng/mL			P
	Initial encounter	Second visit	Third visit	
Time period after the first visit (months)		1.3±0.7	12.2±0.3	
Age				
Mean±SD	21.1±2.8		22.1±2.4	NS
Range	17–29		18–30	
Cycle day at assessment				
Mean±SD	12.6±2.2	2.7±0.9	2.2±1.5	
BMI				
Mean±SD	22.5±4.4	-	22.9±3.2	NS
Cycle length				
Mean±SD	25.1±3.2	-	22.9±2.4	0.01
FSH				
Mean±SD	-	10.9±3.4	12.9±4.7	0.01
LH				
Mean±SD	-	5.9±1.9	8.9±3.9	NS
Estradiol				
Mean±SD	-	52.2±8.3	51.9±25	NS
AMH				
Mean±SD	0.83±0.2	0.78±0.2	0.37±0.1	0.001
AFC				
Mean±SD		2.4±0.9	1.5±0.6	0.001
The number of participants with cycle length≤21 days	6/43 (13.9%)		17/43 (39.5%)	0.013
At least one skipped menses in the last 12 months	0/43 (0%)		6/43 (13.9%)	0.026

FSH: Follicle stimulating hormone

LH: Luteinizing hormone

AMH: Anti-mullerian hormone

AFC: Antral follicle count

BMI: Body mass index

NS: Not significant

**Fig 4:**
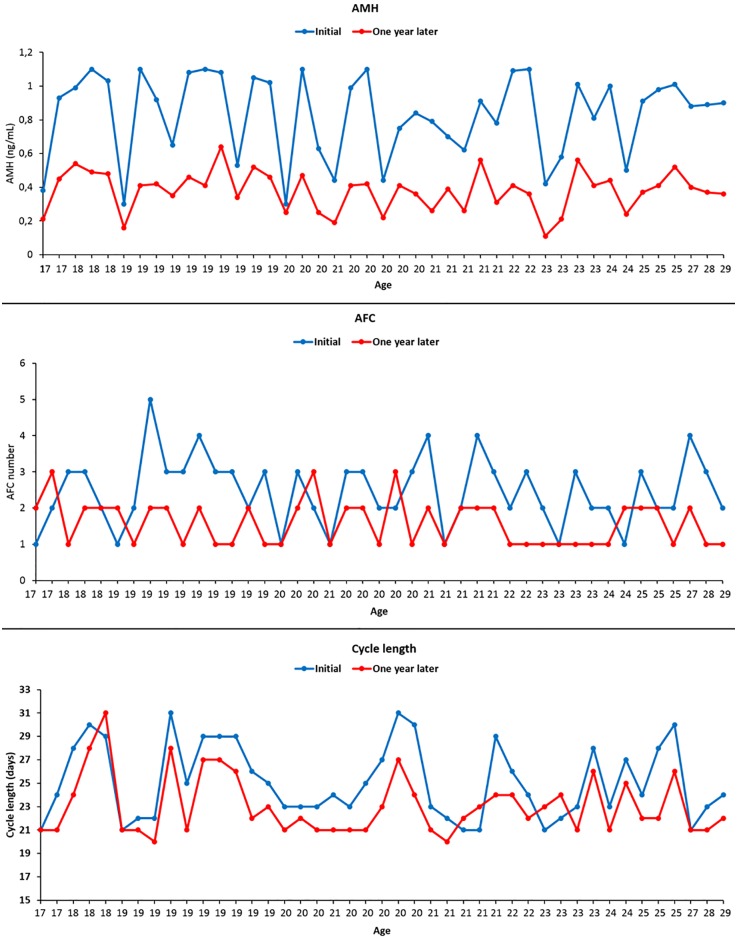
AMH, AFC and cycle length of each participant was reduced at one-year follow-up compared to their baseline values at initial assessment.

The third visit took place 12.2±0.3 months later. All participants returned for this visit. Their repeat hormone tests revealed that AMH (0.37±0.1 vs. 0.83±0.2ng/mL respectively, p<0.001) and AFC (1.6±0.6 vs. 2.4±0.9 respectively, p<0.01) were significantly declined and FSH was significantly elevated (12.9±4.7 vs. 10.9±3.4 mIU/mL respectively, p<0.001) compared to their corresponding values at initial assessment (Table 2, Fig 4). There were no differences in the number of the participants who had undergone TA vs. TV USG between the second and third visits as the only four participants who had had undergone TA USG examination in the second and third visits. Review of the one-year menstrual record charts revealed that the mean cycle length (22.9±2.4 vs. 25.1±3.2 respectively, p<0.01) was significantly shortened and the number of the participants menstruating every 21 days were significantly increased (39.5% vs. 13.9% respectively, p = 0.013) compared to the initial assessment. While none of the participants diagnosed with occult POI had reported any skipped menses in the first assessment, now six of them (13.9%, p = 0.026) reported one missed period at one-year follow-up. Twenty-five participants underwent oocyte freezing. The others did not opt for any fertility preservation method for the following reasons: not interested in childbearing (n = 2), social (n = 8), financial (n = 6), other (n = 2).

## Discussion

We obtained several important findings in this prospective observational study. First, this study confirms one more time that women with critically diminished ovarian reserve may continue to menstruate regularly, emphasizing the fact that we should not rely on regular menses as an indicator of good ovarian reserve. Second, shortening of cycle length precedes menstrual irregularity, making it a more reliable and earlier indicator of diminishing ovarian reserve than any other pattern of menstrual changes in individuals with occult primary ovarian insufficiency (POI). Third, it underscores the importance of screening of young females with possible occult POI with serum AMH measurement as it may develop insidiously in young females with regular menstrual cycle. This is particularly important given that the exact prevalence of occult POI in otherwise healthy young females is largely unknown. It is also unclear if occult POI is precursor of or; distinct from its clinically evident form in terms of prevalence, underlying pathology and natural course. Further, there is no an established method to identify young women with occult POI. Many young women do not regularly visit any health care profession in reproduction especially if they have regular menses and are not interested in childbearing yet. Rather, they are more commonly evaluated by general gynecologists who might not have enough experience and easily overlook low AFC on ultrasound exam that is discordant with patient’s age. Thus, occult POI may remain undiagnosed until it progresses into more advanced stages of low ovarian function and finally become symptomatic as menstrual abnormality and infertility. Even though ESHRE (European Society of Human Reproduction and Embryology) classification of POI did not include AMH as a marker of ovarian reserve, several other studies demonstrated its role in predicting POI in healthy women and Turner’s syndrome patients [13–16]. In this regard, screening of young women with serum AMH measurement might be used to identify them earlier. This was our hypothesis in this study and we demonstrated that forty-three (4.4%) of 963 young females under age 30 have occult POI. Noteworthy, none of these women had any specific menstrual abnormality other than shortening of cycles including those participants with critically diminished ovarian reserve (AMH<0.5ng/mL). At one-year follow-up serum AMH level and AFC were declined and some participants began to experience skipped menses in addition to further shortening of their cycle intervals. Even though long-term follow-up data is not available yet and the findings at one-year follow-up do not fulfill the criteria of POI, these results may at least suggest that occult POI in young females carries the risk of evolving into evident POI.

In contrast to menopause in which permanent cessation of menstruation occurs as a result of depletion of primordial follicle pool, most women with POI may have intermittent and unpredictable ovarian function because the underlying pathology of POI can also be follicle dysfunction in addition to depletion of follicle reserve. The proposed mechanisms of follicle dysfunction in POI include autoimmune lymphocytic oophoritis [17–19]; inappropriate luteinization [5, 20]; or mutation in the FSH receptor gene [21]. POI can also develop as a result of depletion of primordial follicles secondary to accelerated follicle atresia. When this is the case, the underlying pathogenetic mechanism of follicle depletion are heterogeneous ranging from genetic (Turner’s syndrome, mutations in the genes FMR-1, BMP-15, Foxl2, and recently identified MCM9, SYCE1, STAG3) to auto-immune and iatrogenic causes [3]. Unfortunately, we can explain only 10–15% of cases with POI with these mechanisms. The remaining 85–90% appear to have unknown causes. Even though the genes associated with DNA damage and repair are now being increasingly recognized as important genetic mechanisms of POI [22], testing for the mutations of those genes is not indicated routinely for most of them. This is largely because no single genetic mechanism to date has been shown to be a common cause of POI [23]. In fact, it is still unclear whether or not there always has to be an underlying pathology of genetic or other origin to explain every cases of POI? This question is yet to be answered. An interesting study showed us that females might not be born with equal number of primordial follicles in their ovaries [24]. In that study normal human ovaries were collected from 122 women (aged 0–51 years) undergoing elective oophorectomy, organ donation or autopsy. After careful full-thickness sectioning and examination of the whole samples, the authors found out that follicles decay faster with increasing age and there was a considerable percentage of the variation in the number of non-growing follicles (primordial, intermediate and primary) among women that cannot be explained by age alone. More interestingly, great variations were also observed in the follicle counts of newborn ovaries (ranging from 916,000 to 277,000 follicles), challenging our long-held dogmatic presumption that females are born with a set number of follicles (1–2 millions of follicles) in their ovaries (for review; [25]). If this is a generalizable finding, it may explain at least some cases of occult or evident POI because newborn females starting life with less number of follicles in their ovaries might have a shorter reproductive life span and therefore, are more likely to develop POI and premature menopause at an earlier age compared to their age-matched counterparts who harbor more follicles in their gonads.

Pre-mutations in the gene FMR-1 (fragile X mental retardation-1) is well-established cause of primary ovarian insufficiency [26]. There appears to be a non-linear association between the number of repeats and ovarian function. While the carriers of the full mutations do have normal ovarian function those with pre-mutations in the mid-size range seem to have the highest risk of ovarian insufficiency [27, 28]. It was shown by two different groups that FMR-1 pre-mutations are found at increased frequency in women with occult POI [29, 30]. But none of our cases with occult POI had pre-mutation or intermediate alleles in the FMR-1 gene.

Our study has several important limitations. It is unclear whether these findings can be extrapolated to a larger population. Transvaginal ultrasound was not performed in 4 out of 43 (9.3%) patients due to their virginity. Even though these ones constitute only a small fraction of all cases it may limit the reliability of AFC measurements. Early follicular phase FSH measurements may have large inter-cycle variability. Long term follow-up is not available and thus fertility outcome and the timing of menopause cannot be assessed. The ovarian reserve and menstrual characteristics of the participants with AMH>1.1ng/ml were not evaluated one year later. Thus, it is unclear how their ovarian reserve and cycle characteristics changed after one year in comparison to their initial assessment and those with occult POI. Another limitation of our study is that cycle length was reported based on recall at the first screening. There may have been recall bias here. At the second screening, cycle length was reported based on menstruation calendar, thus minimizing recall bias and possibly increasing the number of recalled skipped menses. Besides, these women may have been extra alerted as they were selected to be prospectively followed, possibly creating another recall bias. The threshold AMH value to define occult POI is somewhat arbitrary. It remains to be answered how reliably this cutoff level discriminates women with occult form of POI does from those without it. The ESHRE consensus on the definition of poor responders known as the Bologna criteria considered this cutoff value (0.5–1.1ng/mL) in order to predict the poor response of IVF patients to gonadotropin stimulation based on the area under the curve analyses. It was not aimed to predict reproductive life span of females whose serum AMH are below this threshold level. AMH may exhibit assay-specific pre-analytical instability [31]. Several published studies found that AMH levels obtained with DSL system ELISA kit are significantly lower than the readings of Backman Coulter Gen II assay [32, 33]. The results of these assays are not interchangeable. While the former reports results as nanogram per milliliter (ng/mL), the latter reads as picomol per liter (pmol/L). It was also shown that women with low ovarian reserve may have AMH forms which is not detectable with commercial available assays [34].

## Conclusion

This study underscores the importance of screening young females for possible occult POI as it may develop insidiously without any heralding signs of menstrual abnormality other than shorter cycles. It also appears that occult POI may deteriorate and menstrual abnormalities may begin as early as one year after diagnosis even though it may not fully evolve into clinically evident POI. Declining AMH levels in individuals with occult POI in a relatively short period of time as early as one year as we showed in this study contradicts with the classical description of this clinical entity as a continuum or persistence of impaired ovarian reserve or function. The option of oocyte cryopreservation increased public awareness of ovarian reserve testing in young females who are not currently interested in family building. It may help identify those women with occult POI. However, given that oocyte cryopreservation for social reasons might not be widely available or its regulations by law may change according to the countries, screening of young females with AMH for occult POI might be a valuable tool.

## Supporting information

S1 Table The questionnaire filled by the participants (English version). It includes the questions to gather information about baseline demographic and menstrual characteristics of the participants along with their personal and family history of diseases and surgeries that may affect ovarian reserve and timing of menopause.
(DOCX)

S2 Table The questionnaire filled by the participants (Turkish version). It includes the questions to gather information about baseline demographic and menstrual characteristics of the participants along with their personal and family history of diseases and surgeries that may affect ovarian reserve and timing of menopause.
(DOCX)

S3 Table The menstrual record chart (English version). Every participant diagnosed with occult primary ovarian insufficiency (POI) was requested to keep the records of their one-year menstrual function using this record chart.
(DOCX)

S4 Table The menstrual record chart (Turkish version). Every participant diagnosed with occult primary ovarian insufficiency (POI) was requested to keep the records of their one-year menstrual function using this record chart.
(DOCX)

S1 Information Baseline demographic and menstrual characteristics of the all participants are provided as SPSS data base. (SAV)

S2 Information Baseline demographic and menstrual characteristics of the participants with occult POI are provided as SPSS data base. (SAV)
