# Supplementary material for: Menstrual cycle characteristics of young females with occult primary ovarian insufficiency at initial diagnosis and one-year follow-up with serum amh level and antral follicle count
Source: PLoS One. 2017 Nov 27;12(11):e0188334. doi: 10.1371/journal.pone.0188334 (PMC5703527; doi:10.1371/journal.pone.0188334)
Supplement: S1 Table — It includes the questions to gather information about baseline demographic and menstrual characteristics of the participants along with their personal and family history of diseases and surgeries that may affect ovarian reserve and timing of menopause. (DOCX) [file pone.0188334.s001.docx]

| **QUESTIONNAIRE**  **File Number:**  **Address: Tel:** |
| --- |
| **1. Date of birth: DD/MM/YY** ………………… **2. Height (cm): 3. Weight (kg):** |
| **4. When was your last menstrual period? DD/MM/YY** …………………  **5. How old were you when you started having menstrual periods? ........**  **6. Do you have regular menstrual cycles?** Yes No  If the answer in “No”, please explain in details :  How often have you had menstrual periods in the last year?  Once every 20 days or less Every 21-27 days  Every 28-35 days Every 36-50 days  Every 3-4 months Very irregular, sometimes monthly, sometimes skip several months  Other (Please specify) _______________________________________________________  **7 . The blood sample was taken on which of the menstrual cycle? .............**………….. |
| **8. Have you ever got pregnant?** Yes No  **9. If the answer is yes, how did the pregnancy end?**  Live birth Abortion Stillbirth Termination |
| **11. If you are sexually active, what kind of contraception have you been using: (please skip the question if you are not sexually active)**  **1)** OCP **2)** Condom **3)** IUD **4)** Other (please specify……………) **5)** None |
| **12. Do you have any gynecologic complaints?**  Vaginal discharge Vaginal bleeding Non-menstrual pain  Involuntary passage of urine Pain during urination Frequent urination Galactorrhea Dyspareunia Postcoital bleeding  Pain during menstrual periods Acne Increased hair growth  Spontaneous discharge from nipples  Other (please specify………………) |
| **13. Past medical and surgical history**  **Have you ever seen a medical practitioner about gynecological problems?**  Please specify the diseases you have, or the surgeries you underwent and the medications you have been using including the dates.  Disease:  Surgery:  Medications:  Please explain:  -if you had any gynecologic operations in the past including ovarian surgeries  -if you have an ovarian reserve test (antral follicle count and/or AMH) |
| **14. Family history**  Is there any particular disease running in your family? Yes No  If the answer in “Yes”, please explain in details :  Is there any close family member who had:  Breast cancer:  Gynecological cancers: there any history of premature menopause: (menopause occurring before age 40) in your close family members? Please specify their age at menopause.  Is there any close family member who had premature menopause (<40 years): Yes No  If the answer in “Yes”, please explain in details :  Is there any history of infertility in your first degree relatives? Yes No  If the answer in “Yes”, please explain in details :  Did your mother have any pregnancy complications when she was pregnant to you? Yes No  If the answer in “Yes”, please explain in details :  How did your mother got pregnant to you? Spontaneous In-vitro fertilization |
| **14. Social history**  **Smoking:**  Yes: No. Cigarettes/day: ................ No  **Illicit or re-creational drugs:**  Yes: Please specify: ........................... No  **Alcohol:**  Regular use: Amount /day or night  Social drinker  No **No**  **No**  **Coffee or tea consumption: :**  Yes: No. cups/day: ................ No **Other:** |
| **15. Life style**  **Do you exercise regularly?**  Yes No  If the answer in “Yes”, please explain in details :  **Did you follow any special diet in the last 6 months for weight loss or other purposes?**  Yes No  If the answer in “Yes”, please explain in details : |
| **16. Other**  **Please use this space freely to write if you have anything else to add.** |
