# Supplementary material for: Menstrual cycle characteristics of young females with occult primary ovarian insufficiency at initial diagnosis and one-year follow-up with serum amh level and antral follicle count
Source: PLoS One. 2017 Nov 27;12(11):e0188334. doi: 10.1371/journal.pone.0188334 (PMC5703527; doi:10.1371/journal.pone.0188334)
Supplement: S2 Table — It includes the questions to gather information about baseline demographic and menstrual characteristics of the participants along with their personal and family history of diseases and surgeries that may affect ovarian reserve and timing of menopause. (DOCX) [file pone.0188334.s002.docx]

**TURKISH**

| **ANKET**  **Protokol No:**  **Adres: Tel:** |
| --- |
| **1. Doğum tarihi:Gün/Ay/Yıl** ………………… **2. Boy (cm): 3. Kilo (kg):** |
| **4. Son adet tarihiniz nedir? Gün/Ay/Yıl** …………………  **5. İlk olarak hangi yaşta adet görmeye başladınız?**  **6. Adetleriniz düzenli mi?** Evet Hayır  Eğer cevabınız “Hayır” ise lütfen detaylarıyla açıklayınız: ………………………  Son bir yıl içerisinde hangi aralıklarla adet kanamaları yaşadınız?  20 gün ve daha kısa aralıklarla Her 21-27 günde  Her 28-35 günde Her 36-50 günde  Her 3-4 ayda Çok düzensiz, bazen her ay bazı aylar atlıyor  Diğer (Lütfen açıklayınız) _______________________________________________________  **7. Kan örneği adetin kaçıncı gününde alındı? …………..** |
| **8. Hiç gebelik yaşadınız mı?**  **9. Cevabınız “Evet” ise gebelikleriniz nasıl sonuçlandı? (Sayıları ile belirtiniz?**  Doğum Düşük Ölü doğum Küretaj |
| **11. Cinsel olarak aktif iseniz hangi metod ile gebelikten korunuyorsunuz? (Cinsel hayatınız başlamadıysa lütfen soruyu geçiniz)**  **1)** Doğum kontrol hapı **2)** Kondom **3)** Spiral **4)** Diğer (belirtiniz……………)  **5)** Korunmuyorum |
| **12. Jinekolojik yakınma**  Vajinal akıntı Vajinal kanama Adet dönemi dışında kasık ağrısı  İdrarda yanma İdrar kaçırma Sık idrara gitme  Adet sancıları Akne (Sivilce) Kıllanma  Memeden süt gelmesi  Diabet  Diğer……………… |
| **13. Özgeçmiş**  Hastalık, ilaç kullanımı veya ameliyatınız varsa lütfen belirtiniz.  Hastalık:  Cerrahi:  İlaç kullanımı:  Lütfen açıklayınız eğer:  -Yumurtalıklarınıza içeren veya içermeyen bir jinekolojik cerrahi veya hastalık geçirdiyseniz  -AMH ve/veya antral folikül sayısı belirten yumurtalık rezerv testininz varmı? |
| **14. Aile öyküsü**  Ailenizde kalıtsal geçiş gösteren bir hastalık var mı? Evet Hayır  Cevabınız “Evet” ise lütfen detaylarıyla açıklayınız: ………………………  Yakın akrabalarınız arasında  Meme kanseri:  Jinekolojik kanserler:  Ailenizde erken menapoz (40 yaş öncesi) var mı? Evet Hayır  Cevabınız “Evet” ise lütfen detaylarıyla açıklayınız: ………………………  Ailenizde infertilite problemi yaşayan var mı? Evet Hayır  Cevabınız “Evet” ise lütfen detaylarıyla açıklayınız: ………………………  Annenizin size hamile iken geçirdiği bir hastalık var mı? Evet Hayır  Cevabınız “Evet” ise lütfen detaylarıyla açıklayınız: ………………………  Annenizin size hamileliği nasıl gerçekleşmiş? Kendiliğinden Tüp bebek tedavisiyle |
| **14. Alışkanlıklar**  **Sigara:**  Evet, Sigara/gün: ........ Hayır  **Uyuşturucu :**  Evet: Lütfen açıklayınız: ........................... Hayır  **Alkol:**  Düzenli kullanım: Bardak/gün .................  Sosyal içici  Hayır  **Kahve/çay:**  Evet fincan/gün: ................ Hayır |
| **15. Hayat stili**  **Düzenli egzersiz yapar mısınız?**  Evet Hayır  Cevabınız “Evet” ise lütfen detaylarıyla açıklayınız: ………………………  **Son 6 ay içinde özel bir diyet uyguladınız mı?**  Cevabınız “Evet” ise lütfen detaylarıyla açıklayınız: ……………………… |
| **16. Diğer**  **Ekleyeceklerinizi açık bir şekilde belirtiniz. Teşekkürler.** |
