# Supplementary material for: Menstrual cycle characteristics of young females with occult primary ovarian insufficiency at initial diagnosis and one-year follow-up with serum amh level and antral follicle count
Source: PLoS One. 2017 Nov 27;12(11):e0188334. doi: 10.1371/journal.pone.0188334 (PMC5703527; doi:10.1371/journal.pone.0188334)
Supplement: S4 Table — Every participant diagnosed with occult primary ovarian insufficiency (POI) was requested to keep the records of their one-year menstrual function using this record chart. (DOCX) [file pone.0188334.s004.docx]

Menstruasyon kanaması günlüğü

Protokol numarası:

Yıl:

| Ay | Gün | | | | | | | | | | | | | | | | | | | | | | | | | | | | | | | Bir adet kanamasının ilk gününden diğerine kadar geçen süre (gün) |
| --- | --- | --- | --- | --- | --- | --- | --- | --- | --- | --- | --- | --- | --- | --- | --- | --- | --- | --- | --- | --- | --- | --- | --- | --- | --- | --- | --- | --- | --- | --- | --- | --- |
|  | 1 | 2 | 3 | 4 | 5 | 6 | 7 | 8 | 9 | 10 | 11 | 12 | 13 | 14 | 15 | 16 | 17 | 18 | 19 | 20 | 21 | 22 | 23 | 24 | 25 | 26 | 27 | 28 | 29 | 30 | 31 |  |
| Ocak |  |  |  |  |  |  |  |  |  |  |  |  |  |  |  |  |  |  |  |  |  |  |  |  |  |  |  |  |  |  |  |  |
| Şub |  |  |  |  |  |  |  |  |  |  |  |  |  |  |  |  |  |  |  |  |  |  |  |  |  |  |  |  |  |  |  |  |
| Mar |  |  |  |  |  |  |  |  |  |  |  |  |  |  |  |  |  |  |  |  |  |  |  |  |  |  |  |  |  |  |  |  |
| Nis |  |  |  |  |  |  |  |  |  |  |  |  |  |  |  |  |  |  |  |  |  |  |  |  |  |  |  |  |  |  |  |  |
| May |  |  |  |  |  |  |  |  |  |  |  |  |  |  |  |  |  |  |  |  |  |  |  |  |  |  |  |  |  |  |  |  |
| Haz |  |  |  |  |  |  |  |  |  |  |  |  |  |  |  |  |  |  |  |  |  |  |  |  |  |  |  |  |  |  |  |  |
| Tem |  |  |  |  |  |  |  |  |  |  |  |  |  |  |  |  |  |  |  |  |  |  |  |  |  |  |  |  |  |  |  |  |
| Ağus |  |  |  |  |  |  |  |  |  |  |  |  |  |  |  |  |  |  |  |  |  |  |  |  |  |  |  |  |  |  |  |  |
| Eyl |  |  |  |  |  |  |  |  |  |  |  |  |  |  |  |  |  |  |  |  |  |  |  |  |  |  |  |  |  |  |  |  |
| Ekim |  |  |  |  |  |  |  |  |  |  |  |  |  |  |  |  |  |  |  |  |  |  |  |  |  |  |  |  |  |  |  |  |
| Kas |  |  |  |  |  |  |  |  |  |  |  |  |  |  |  |  |  |  |  |  |  |  |  |  |  |  |  |  |  |  |  |  |
| Ara |  |  |  |  |  |  |  |  |  |  |  |  |  |  |  |  |  |  |  |  |  |  |  |  |  |  |  |  |  |  |  |  |

Lütfen doktorunuz ile görüşürken bu kaydı yanınızda bulundurunuz.

Menstruasyon kanamasının miktarı: X: Normal, S: Hafif, H: Yoğun

Menstruasyon kanamasının her günü için kullanılan hijyenik pet sayısını ve Petlerin kan ile boyanmasının tarzını belirtiniz.

F: Tamamen, P:Kısmen , S:Lekelenme

Ör: X/P
